# Levy Noise Affects Ornstein–Uhlenbeck Memory

**DOI:** 10.3390/e27020157

**Published:** 2025-02-02

**Authors:** Iddo Eliazar

**Affiliations:** School of Chemistry, Tel Aviv University, Tel Aviv 6997801, Israel; eliazar@tauex.tau.ac.il

**Keywords:** Ornstein–Uhlenbeck process, Langevin equation, memory, Gauss and Levy noises, light and heavy tails, short-range and long-range correlations, Noah and Joseph effects, 02.50.-r (probability theory, stochastic processes, and statistics), 05.40.-a (fluctuation phenomena, random processes, noise, and Brownian motion)

## Abstract

This paper investigates the memory of the Ornstein–Uhlenbeck process (OUP) via three ratios of the OUP increments: signal-to-noise, noise-to-noise, and tail-to-tail. Intuition suggests the following points: (1) changing the noise that drives the OUP from Gauss to Levy will not affect the memory, as both noises share the common ‘independent increments’ property; (2) changing the auto-correlation of the OUP from exponential to slowly decaying will affect the memory, as the change yields a process with long-range correlations; and (3) with regard to Levy driving noise, the greater the noise fluctuations, the noisier the prediction of the OUP increments. This paper shows that intuition is plain wrong. Indeed, a detailed analysis establishes that for each of the three above-mentioned points, the very converse holds. Hence, Levy noise has a significant and counter-intuitive effect on Ornstein–Uhlenbeck memory.

## 1. Introduction

For random processes that are in statistical equilibria, the foundational ‘go-to’ stochastic model is the *Ornstein–Uhlenbeck process* (OUP) [1,2,3,4]. Mathematically, the OUP is the intersection of three principal classes of random processes [5]: Gaussian [6,7,8]; stationary [9,10,11]; and Markovian [12,13,14]. Being a Gaussian and stationary random process, the OUP is characterized by its auto-correlation function, which is exponential. The dynamics of the OUP are governed by the *Langevin equation* [15,16,17]. In addition, the stochastic ‘engine’ that ‘drives’ the OUP is Gauss white noise (GWN), which is the velocity process of Brownian motion [18].

The literature on the OUP is vast, e.g., [2,3,4,19,20,21,22,23,24,25,26]. Recent examples of OUP research include: active Ornstein–Uhlenbeck particles [27,28,29,30,31]; OUP and stochastic resetting [32,33,34]; OUP parameter estimation [35,36]; OUP time averages [37]; phase descriptions of multidimensional OUP [38]; OUP and ergodicity breaking [39]; OUP survival analysis [40]; OUP first-passage area [41]; OUP and optical tweezers [42]; OUP large deviations [43]; high-dimensional OUP [44]; and OUP with a memory kernel [45].

The OUP is a ‘regular’ stochastic model. On the one hand, being a Gaussian process, the OUP belongs to the realm of finite-variance and light-tailed stochastic models. On the other hand, as its exponential auto-correlation function decays rapidly, the OUP belongs to the realm of stochastic models with short-range correlations. There are two main approaches that turn the OUP from a regular to an ‘anomalous’ stochastic model: Levy and Gauss.

The Levy approach shifts the OUP (from the realm of finite-variance and light-tailed stochastic models) to the realm of infinite-variance and heavy-tailed stochastic models [46,47]; Mandelbrot and Wallis termed this shift the “Noah effect” [48]. Specifically, the Levy approach replaces the GWN that drives the OUP with Levy noise, which is the velocity process of the (symmetric and stable) Levy motion [49,50,51].

This replacement yields the *Levy-driven OUP* [52,53,54,55,56,57,58,59,60,61,62,63] and the *Levy-driven Langevin equation* [64,65,66,67,68,69,70,71,72,73,74,75]. Applications of the Levy-driven OUP include telecommunications [76], reliability analysis [77], fuel switching [78], insurance [79], and finance [80,81,82].

Stochastic models that are driven by Levy noise attracted major interest in statistical physics [83,84,85,86,87,88,89,90,91,92,93,94]. In particular—as evident from the above references—so did the Levy-driven OUP and the Levy-driven Langevin equation. As evident from the following recent research examples, interest in general Levy-driven models [95,96,97,98,99,100,101,102,103,104,105,106,107,108,109,110,111], Levy-driven OUP [79,82,112,113,114,115], and the Levy-driven Langevin equation [116,117,118,119,120,121,122] is ongoing.

The Gauss approach shifts the OUP (from the realm of stochastic models with short-range correlations) to the realm of stochastic models with long-range correlations [123,124,125]; Mandelbrot and Wallis termed this shift the “Joseph effect” [48]. Specifically, the Gauss approach replaces the OUP’s exponential auto-correlation function with auto-correlation functions that decay slowly.

Both approaches maintain the stationary property of the OUP. With regard to the Gaussian and Markovian properties of the OUP, the approaches act in ‘orthogonal directions’. The Levy approach maintains the Markovian property, and it discards the Gaussian property. Conversely, the Gauss approach (as its name suggests) maintains the Gaussian property, and it discards the Markovian property.

GWN and Levy noise share a common independence property: their noise values, at different time points, are statistically independent (a precise definition of the independence property will be stated in Section 2.3). Consequently, one would expect that the Levy approach will not affect process memory. On the other hand—due to the shift from short-range to long-range correlations—one would expect that the Gauss approach will affect process memory. This paper establishes that it is actually the very converse that holds: **the Levy approach yields memory effects which the Gauss approach does not**.

To attain its aim, the paper advances as follows. The increment of a random process is its displacement over a specified temporal interval. The increment’s conditional distribution is that in which the following information is provided: the position of the random process at the initial time point of the increment’s temporal interval. Based on the increment’s unconditional and conditional statistical distributions, three informative ratios are devised and analyzed: a ‘signal-to-noise’ ratio; a ‘noise-to-noise’ ratio; and a ‘tail-to-tail’ ratio. These ratios quantify process memory, and their analysis establishes the following conclusions:

▶ The Levy approach yields memory effects that are markedly more profound than those of the OUP.
▶ The Gauss approach yields memory effects that are qualitatively identical to those of the OUP.

In addition to the above conclusions, yet another counter-intuitive conclusion is reached. Indeed, with regard to the Levy-driven OUP, consider the prediction of the above increment, provided the above information. One would expect that the greater the fluctuations in the driving Levy noise, the more ‘noisy’ the prediction. Further analysis of the noise-to-noise ratio establishes that it is actually the very converse that holds: **the greater the fluctuations in the driving Levy noise, the less noisy the prediction**.

The paper is organized as follows: Section 2 ‘sets the stage’ with concise reviews of several notions (that underpin this research). Section 3 presents the unconditional and conditional statistical distributions of the Levy-driven OUP increments. Section 4 devises the three ratios, and analyzes them for the Levy approach. Section 5 analyzes the three ratios for the Gauss approach. Lastly, Section 6 concludes with a discussion that compares the Levy approach to the Gauss approach, and that describes the counter-intuitive nature of the key results established here.

**Future research** can proceed in two possible directions. On the one hand, theoreticians can look for additional counter-intuitive behaviors of the Levy-driven OUP. On the other hand, practitioners can look for ‘real-world’ applications that exploit the counter-intuitive behaviors of the Levy-driven OUP.

## 2. Setting the Stage

This section sets the stage for the topic of this paper. The section tersely reviews the following notions: the measurement of randomness (Section 2.1); Levy distributions (Section 2.2); Levy noise (Section 2.3); the Levy-driven Langevin equation and the Ornstein–Uhlenbeck process (Section 2.4).

### 2.1. Measuring Randomness

Consider a real-valued random variable *X*. The most common way to quantify the randomness of *X* is via its standard deviation, σ[X], which displays the two following *gauge properties*.

**G1.**  The standard deviation is non-negative, σ[X]≥0, and it vanishes if and only if the random variable is deterministic:
  (1) σ[X]=0⇔X=const,
  where the equality on the right-hand side holds with probability one.
**G2.**  The standard-deviation’s response to an affine transformation of the random variable is:
  (2) σ[aX+b]=|a|·σ[X],
  where *a* and *b* are, respectively, the slope and the intercept of the affine transformation.

In fact, *any* gauge σ[X] that displays the two gauge properties is a legitimate measure of randomness [126,127]. For example, the perplexity [128]—which is the exponentiation of Shannon entropy [129]—is a measure of randomness. The inverse participation ratio [130,131]—which is the exponentiation of the collision entropy [132,133]—is also a measure of randomness. The reciprocal of the inverse participation ratio is known as Simpson’s index in biology and ecology [134], and as Hirschman’s index in economics and competition law [135]. The perplexity and the inverse participation ratio are special cases of yet another measure of randomness—the diversity [136,137,138,139,140], which is the exponentiation of Renyi entropy [141].

### 2.2. Levy Distribution

As above, consider a real-valued random variable *X*. The statistical distribution of *X* is *symmetric Levy-stable* [142,143,144] when

(3) $$E\left[\exp\left(i\theta X\right)\right]=\exp\left(ic\theta -\frac{1}{p}{\left|s\theta \right|}^{p}\right)$$

(−∞<θ<∞). Namely, Equation (3) is the characteristic function of the random variable *X*, and it has three parameters: a real center parameter *c*; a positive scale parameter *s*; and a power *p* whose admissible values are 0<p≤2.

Henceforth, a random variable *X*—whose characteristic function is that of Equation (3)—is termed, in short, *Levy*. In turn, the power *p* is henceforth termed *Levy exponent*. Key facts regarding the Levy random variable *X* are the following [142,143,144]:

**F1.**  The random variable *X* is symmetric about its center *c*, and hence: the median of *X* is its center, M[X]=c.
**F2.**  When the Levy exponent is in the range 0<p≤1 then the mean absolute deviation of *X* from its center diverges: $E\left[\left|X-c\right|\right]=\infty$.
**F3.**  When the Levy exponent is one, p=1, then the statistical distribution of *X* is *Cauchy*.
**F4.**  When the Levy exponent is in the range 1<p<2 then: the mean of *X* is its center, E[X]=c; and the mean squared deviation of *X* from its center diverges, $E[{\left|X-c\right|}^{2}]=\infty$.
**F5.**  When the Levy exponent is two, p=2, then the statistical distribution of *X* is *Gauss*, and hence: the parameters *c* and *s* are, respectively, the mean and the standard deviation of *X*.

The facts stated above imply that the mean behavior of the Levy random variable *X* changes dramatically as the Levy exponent *p* crosses the ‘Cauchy threshold’ p=1. This threshold shall appear time and again along this paper.

The standard deviation of the Levy random variable *X* is well defined only in the Gauss case (p=2). Other measures of randomness—e.g., the ones noted in Section 2.1—are well defined at large. For a measure of randomness which is well defined, Equations (2) and (3) imply that

(4) $$\sigma \left[X\right]=\sigma_{p}\cdot s\;,$$

where $\sigma_{p}$ is the measure of randomness of a ‘normalized’ Levy random variable. Namely, a Levy random variable with center zero c=0 and with scale one s=1. So, up to a coefficient, the scale *s* of the Levy random variable *X* manifests its randomness.

### 2.3. Levy Noise

Consider a real-valued stochastic process $\left\{\eta \left(u\right);-\infty <u<\infty \right\}$. Namely, the process is defined over the real line (−∞<u<∞), and its values are $\eta \left(u\right)$. The process is a *Levy noise* when it displays the two following *Levy properties* [49,50,51].

**L1.**  The integral of the noise over a time interval of duration Δ is a Levy random variable with center zero c=0 and scale $s=\Delta^{1/p}$ (where *p* is the Levy exponent).
**L2.**  The integrals of the noise over disjoint time intervals are independent random variables.

The Levy noise admits a characterization in terms of its integrals with respect to ‘test functions’: $\int_{-\infty }^{\infty }f\left(u\right)\eta \left(u\right)du$, where $f\left(u\right)$ is a general test function. Indeed, the process is a Levy noise if and only if the integral is a Levy random variable with center zero, and with scale

(5) $${∥f∥}_{p}:={\left[\int_{-\infty }^{\infty }{\left|f\left(u\right)\right|}^{p}du\right]}^{1/p}\;.$$

The quantity appearing in Equation (5) is the “$L_{p}$ norm” of the test function $f\left(u\right)$ (with respect to the Lebesgue measure over the real line). The Levy exponent two, p=2, characterizes a special case in which: the Levy noise is Gauss white noise (GWN), the velocity process of Brownian motion [18]; and the corresponding $L_{2}$ norm is the Hilbert norm.

The behavior of the $L_{p}$ norm changes dramatically as the Levy exponent *p* crosses the Cauchy threshold p=1 (which was noted in Section 2.2). Indeed, consider the unit ball with respect to the $L_{p}$ norm, i.e.: the collection of all test functions $f\left(u\right)$ with a norm that is no larger than one, ${∥f∥}_{p}\leq 1$. This unit ball is *not* convex when the Levy exponent is in the range 0<p<1, and it *is* convex when the Levy exponent is in the range 1≤p≤2.

### 2.4. Langevin, Ornstein and Uhlenbeck

Arguably, the best known ordinary differential equation is the linear one. In turn, adding noise to the linear ordinary differential equation yields what is arguably the best known stochastic differential equation—the *Langevin equation* [15,16,17]—which is described as follows.

Consider a real-valued random motion $\left\{X\left(t\right);-\infty <t<\infty \right\}$. Namely, the underlying timeline is −∞<t<∞, and the motion’s position at the time point *t* is $X\left(t\right)$. The motion’s dynamics are governed by the Langevin equation when

(6) $$\dot{X}\left(t\right)=-\alpha X\left(t\right)+\beta \eta \left(t\right)\;,$$

where: α is a positive damping coefficient; β is a positive noise coefficient; and $\left\{\eta \left(t\right);-\infty <t<\infty \right\}$ is a real-valued noise that ‘drives’ the Langevin equation.

Integrating the Langevin Equation (6), while setting the integration constant to be zero, yields the following integral representation of the motion’s positions:

(7) $$X\left(t\right)=\underset{D\left(t\right)}{\underset{︸}{\beta \exp\left(-\alpha t\right)}}\cdot \underset{I\left(t\right)}{\underset{︸}{\int_{-\infty }^{t}\exp\left(\alpha u\right)\eta \left(u\right)du}\;}.$$

Namely, the integral representation of Equation (7) is the product of two terms—one deterministic and one stochastic. The deterministic term, $D\left(t\right)$, is an exponential decay function. The stochastic term, $I\left(t\right)$, is a ‘running integral’ of the noise with respect to an exponential growth function.

Traditionally, the noise that drives the Langevin equation is set to be GWN. In turn, the resulting motion is the *Ornstein–Uhlenbeck process* (OUP) [1,2,3,4]. As noted in Section 2.3, GWN is the Levy noise with the Levy exponent two, p=2. In general, when the noise is Levy then: Equation (6) is the *Levy-driven Langevin equation* [64,65,66,67,68,69,70,71,72,73,74,75]; and Equation (7) is an integral representation of the *Levy-driven OUP* [52,53,54,55,56,57,58,59,60,61,62,63].

## 3. Increments of the Levy-Driven OUP

This section sets the focus on the increments of the Levy-driven OUP. The positions of the process admit the integral representation of Equation (7). The increment of the process over the time interval (t,t+Δ)—where *t* is a real time point, and where Δ is a positive duration—is the displacement $X\left(t+\Delta \right)-X\left(t\right)$. The increments’ unconditional and conditional statistics will be addressed, respectively, in Section 3.1 and Section 3.2.

### 3.1. Increments’ Unconditional Statistics

Equation (7) implies that the increment of the process over the time interval (t,t+Δ) admits the following integral representation:

(8) $$\left.\begin{array}{c} X\left(t+\Delta \right)-X\left(t\right)=D\left(t+\Delta \right)I\left(t+\Delta \right)-D\left(t\right)I\left(t\right)\\ \;\\ =\left[D\left(t+\Delta \right)-D\left(t\right)\right]I\left(t\right)+D\left(t+\Delta \right)\left[I\left(t+\Delta \right)-I\left(t\right)\right]\\ \;\\ =\int_{-\infty }^{t}\left[D\left(t+\Delta \right)-D\left(t\right)\right]\exp\left(\alpha u\right)\eta \left(u\right)du\\ \;\\ \;\;\;\;\;+\int_{t}^{t+\Delta }D\left(t+\Delta \right)\exp\left(\alpha u\right)\eta \left(u\right)du\;. \end{array}\right.$$

So, the increment admits the formulation $X\left(t+\Delta \right)-X\left(t\right)=\int_{-\infty }^{\infty }f\left(u\right)\eta \left(u\right)du$, where: (**i**) $f\left(u\right)=\left[D\left(t+\Delta \right)-D\left(t\right)\right]\exp\left(\alpha u\right)$ when u≤t; (**ii**) $f\left(u\right)=D\left(t+\Delta \right)\exp\left(\alpha u\right)$ when t<u≤t+Δ; and (**iii**) $f\left(u\right)=0$ when u>t+Δ.

In turn, it follows from Section 2.3 that the increment $X\left(t+\Delta \right)-X\left(t\right)$ is a Levy random variable with center zero, and with scale

(9) $$\left.\begin{array}{c} {∥f∥}_{p}=\left\{\int_{-\infty }^{t}{\left|\left[D\left(t+\Delta \right)-D\left(t\right)\right]\exp\left(\alpha u\right)\right|}^{p}du\right.\\ \;\\ \;\;\;\;\;{\left.+\int_{t}^{t+\Delta }{\left|D\left(t+\Delta \right)\exp\left(\alpha u\right)\right|}^{p}du\right\}}^{1/p}\\ \;\\ =\frac{\beta }{{\left(\alpha p\right)}^{1/p}}{\left\{{\left[1-\exp\left(-\alpha \Delta \right)\right]}^{p}+\left[1-\exp\left(-\alpha p\Delta \right)\right]\;\right\}}^{1/p}\;. \end{array}\right.$$

The transition from the top line of Equation (9) to its bottom line is due to a straightforward calculation.

### 3.2. Increments’ Conditional Statistics

The Equations (7) and (8) imply that

(10) $$\left.\begin{array}{c} X\left(t+\Delta \right)-X\left(t\right)=\left[\frac{D\left(t+\Delta \right)}{D\left(t\right)}-1\right]X\left(t\right)\\ \;\\ \;\;\;\;\;\;\;\;\;\;+\int_{t}^{t+\Delta }D\left(t+\Delta \right)\exp\left(\alpha u\right)\eta \left(u\right)du\;. \end{array}\right.$$

Also, Equation (7) and the properties of the Levy noise imply that the position $X\left(t\right)$ and the integral appearing on the right-hand side of Equation (10) are independent random variables.

Given the information $X\left(t\right)$, the increment $X\left(t+\Delta \right)-X\left(t\right)$ has two parts: deterministic and stochastic. The deterministic part is the term, on the right-hand side of Equation (10), that involves $X\left(t\right)$. The stochastic part is the integral on the right-hand side of Equation (10). Note that the integral admits the formulation $\int_{-\infty }^{\infty }f\left(u\right)\eta \left(u\right)du$, where: (**i**) $f\left(u\right)=0$ when u≤t; (**ii**) $f\left(u\right)=D\left(t+\Delta \right)\exp\left(\alpha u\right)$ when t<u≤t+Δ; and (**iii**) $f\left(u\right)=0$ when u>t+Δ.

So, given the information $X\left(t\right)$, the conditional distribution of the increment $X\left(t+\Delta \right)-X\left(t\right)$ is Levy with the following center and scale parameters. The center of the conditional distribution is the deterministic part

(11) $$\left[\frac{D\left(t+\Delta \right)}{D\left(t\right)}-1\right]X\left(t\right)=-\left[1-\exp\left(-\alpha \Delta \right)\right]X\left(t\right)\;.$$

The scale of the conditional distribution is that of the stochastic part

(12) $$\left.\begin{array}{c} {∥f∥}_{p}={\left\{\int_{t}^{t+\Delta }{\left|D\left(t+\Delta \right)\exp\left(\alpha u\right)\right|}^{p}du\;\right\}}^{1/p}\\ \;\\ =\frac{\beta }{{\left(\alpha p\right)}^{1/p}}{\left[1-\exp\left(-\alpha p\Delta \right)\;\right]}^{1/p}\;. \end{array}\right.$$

The transition from the top line of Equation (12) to its bottom line is due to a straightforward calculation.

## 4. Three Ratios

With regard to the unconditional and conditional statistical distributions of the increment $X\left(t+\Delta \right)-X\left(t\right)$ (which were presented in Section 3), this section will devise and analyze three informative ratios: a ‘signal-to-noise’ ratio (Section 4.1); a ‘noise-to-noise’ ratio (Section 4.2); and a ‘tail-to-tail’ ratio (Section 4.3). The three ratios will involve the function

(13) $$\phi_{p}\left(v\right)=\frac{{\left[1-\exp\left(-v\right)\right]}^{p}}{1-\exp\left(-pv\right)}\;,$$

where: the function’s variable *v* is positive (0<v<∞); and the function’s parameter *p* is the Levy exponent (0<p≤2). The properties of the function $\phi_{p}\left(v\right)$, which will be used along this section, are derived in Appendix A.

### 4.1. Signal-to-Noise Ratio

Consider a statistical distribution of interest, which has a real mean and a positive standard deviation. The distribution’s *signal-to-noise ratio* is that of the following quantities: the numerator is the absolute value of the distribution’s mean; and the denominator is the distribution’s standard deviation. As its name suggests, the ratio measures how strong the distribution’s ‘signal’ (i.e., mean) is—measured relative to the distribution’s ‘noise’ (i.e., standard deviation).

When the distribution of interest is Levy, then the mean is well-defined only when the Levy exponent is in the range 1<p≤2, and the standard deviation is positive only when the Levy exponent is p=2. So, albeit in the Gauss case (p=2), the signal-to-noise ratio cannot be applied ‘as is’ to the Levy distribution.

Nonetheless, with some ‘tinkering’, the signal-to-noise ratio can be modified to fit the Levy distribution. Indeed, on the one hand, we can replace the mean with the median—which is well defined, and which coincides with the mean when the latter is well defined (1<p≤2). On the other hand, we can replace the standard deviation with a measure of randomness σ[·] that is well defined for the Levy distribution (e.g., the above-mentioned perplexity, inverse participation ratio, and diversity).

Replacing the mean with the median is akin—in the context of signal processing—to replacing moving-average filters with median filters [145,146,147]. Also, replacing the standard deviation with other measures of randomness is akin—in the context of financial markets—to replacing volatility with entropy-based measures [148,149,150].

As shown in Section 3.1, the increment’s unconditional distribution is Levy with a zero center. In turn, the modified signal-to-noise ratio of the increment’s unconditional distribution is zero. Matters change dramatically when switching from the increment’s unconditional distribution to its conditional distribution.

As shown in Section 3.2, the increment’s conditional distribution is Levy, with a center specified in Equation (11) and a scale specified in Equation (12). In turn, the fact **F1** of Section 2.2 and Equation (4) implies that the modified signal-to-noise ratio of the increment’s conditional distribution is:

(14) $$\frac{\left|M\left[X\left(t+\Delta \right)-X\left(t\right)|X\left(t\right)\right]\right|}{\sigma \left[X\left(t+\Delta \right)-X\left(t\right)|X\left(t\right)\right]}=\underset{\omega }{\underset{︸}{\left[\frac{{\left(\alpha p\right)}^{1/p}}{\beta \sigma_{p}}\left|X\left(t\right)\right|\right]}}\cdot \phi_{p}{\left(\alpha \Delta \right)}^{1/p}\;,$$

where the coefficient $\sigma_{p}$ is that of Equation (4). Note that the particular choice of the measure of randomness σ[·] affects the signal-to-noise ratio of Equation (14) only via the coefficient $\sigma_{p}$.

It follows straightforwardly from Equation (13) that $\lim_{v\to \infty }\phi_{p}\left(v\right)=1$, and that: when the Levy exponent is p=1 then the function is flat, $\phi_{1}\left(v\right)=1$. It is shown in Appendix A that the shape of the function $\phi_{p}\left(v\right)$ is determined by the Levy exponent *p* as follows. When the Levy exponent is in the range 0<p<1 then: $\lim_{v\to 0}\phi_{p}\left(v\right)=\infty$, and the function is monotone decreasing. When the Levy exponent is in the range 1<p≤2 then: $\lim_{v\to 0}\phi_{p}\left(v\right)=0$, and the function is monotone increasing.

**Figure 1:**
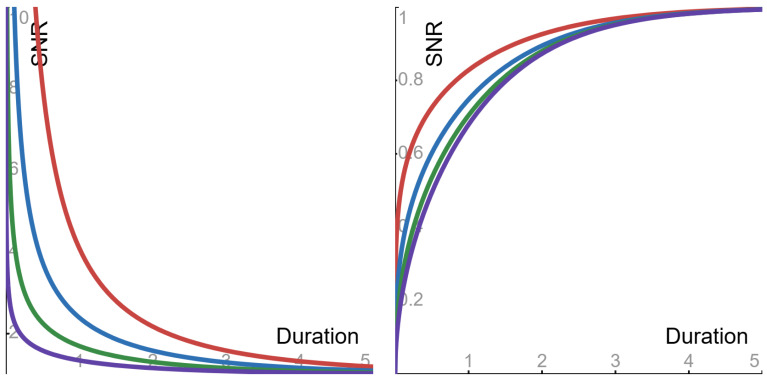
The signal-to-noise ratio (SNR) of Equation (14), as a function of the duration Δ (with parameters α = 1 and ω = 1). Sub-Cauchy examples are depicted in the **left panel**, with the following Levy exponents: red 0.5; blue 0.6; green 0.7; purple 0.8. Super-Cauchy and Gauss examples are depicted in the **right panel**, with the following Levy exponents: red 1.25; blue 1.5; green 1.75; purple 2.

Consider the given information not to be zero, $X\left(t\right)\neq 0$. Due to the properties of the function $\phi_{p}\left(v\right)$, the asymptotic value—as Δ→∞—of the signal-to-noise ratio of Equation (14) is the positive number ω. Also—as a function of the duration Δ—the signal-to-noise ratio of Equation (14) displays the following behaviors (see Figure 1).

▶ **Sub-Cauchy** (0<p<1) **case**: the ratio is *monotone decreasing* from *∞* to its asymptotic value.
▶ **Cauchy** (p=1) **case**: the ratio is *flat*, and its constant value is its asymptotic value.
▶ **Super-Cauchy** (1<p<2) and **Gauss** (p=2) **cases**: the ratio is *monotone increasing* from 0 to its asymptotic value.

### 4.2. Noise-to-Noise Ratio

As in Section 4.1, consider a measure of randomness σ[·] that is well defined for the Levy distribution. The *noise-to-noise ratio* of the increment $X\left(t+\Delta \right)-X\left(t\right)$ is that of the following quantities: the numerator is the measure of randomness of the increment’s conditional distribution; and the denominator is the measure of randomness of the increment’s unconditional distribution.

Equation (4) implies that the noise-to-noise ratio is a scale-to-scale ratio—that of the scale of the increment’s conditional distribution to the scale of the increment’s unconditional distribution. These scales are specified in Equations (9) and (12), and (after a bit of algebra) they yield the ratio

(15) $$\frac{\sigma \left[X\left(t+\Delta \right)-X\left(t\right)|X\left(t\right)\right]}{\sigma \left[X\left(t+\Delta \right)-X\left(t\right)\right]}=\frac{1}{{\left[1+\phi_{p}\left(\alpha \Delta \right)\right]}^{1/p}}\;.$$

The noise-to-noise ratio of Equation (15) is *universal*: as it is a scale-to-scale ratio—it is invariant with respect to the particular choice of the measure of randomness σ[·] (that is well defined for the Levy distribution).

The noise-to-noise ratio of Equation (15) quantifies the extent to which the given information $X\left(t\right)$ reduces the measured randomness of the increment $X\left(t+\Delta \right)-X\left(t\right)$. This ratio takes values in the unit interval: it is bounded from below by the value zero—which manifests full (100%) reduction in randomness; and it is bounded from above by the value one—which manifests no (0%) reduction in randomness.

**Figure 2:**
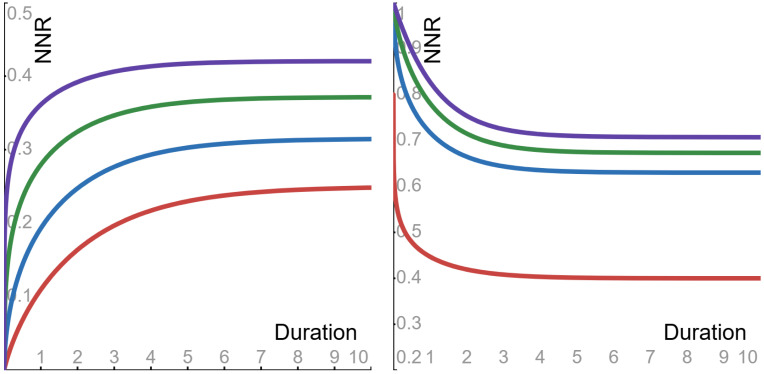
The noise-to-noise ratio (NNR) of Equation (15), as a function of the duration Δ (with parameter α = 1). Sub-Cauchy examples are depicted in the **left panel**, with the following Levy exponents: red 0.5; blue 0.6; green 0.7; purple 0.8. Super-Cauchy and Gauss examples are depicted in the **right panel**, with the following Levy exponents: red 1.25; blue 1.5; green 1.75; purple 2.

The properties of the function $\phi_{p}\left(v\right)$ were described in Section 4.1. Due to these properties, the asymptotic value—as Δ→∞—of the noise-to-noise ratio of Equation (15) is $1/2^{1/p}$. Also—as a function of the duration Δ—the noise-to-noise ratio of Equation (15) displays the following behaviors (see Figure 2).

▶ **Sub-Cauchy** (0<p<1) **case**: the ratio is *monotone increasing* from 0 to its asymptotic value.
▶ **Cauchy** (p=1) **case**: the ratio is *flat*, and its constant value is its asymptotic value.
▶ **Super-Cauchy** (1<p<2) and **Gauss** (p=2) **cases**: the ratio is *monotone decreasing* from 1 to its asymptotic value.

As noted in Section 2.4, the parameter α is the damping coefficient of the Langevin Equation (6), and it is positive. It follows straightforwardly from Equation (15) that the monotonicity behavior of the noise-to-noise ratio—with respect to the duration Δ—holds identically also with respect to the parameter α.

### 4.3. Tail-to-Tail Ratio

As in Section 2.2, consider a real-valued random variable *X* whose statistical distribution is Levy with center *c*, scale *s*, and Levy exponent *p*. In the Gauss case (p=2), the probability tails of random variable *X* are ‘light’, i.e., they exhibit a super-exponential decay. In sharp contrast, when the Levy exponent is in the range 0<p<2, then the probability tails of random variable *X* are ‘heavy’ [46,47]. Specifically, the heavy probability tails exhibit the following power-law decay [142,143,144]:

(16) $$\mathrm{Pr}\left(\left|X-c\right|>x\right)\approx \tau_{p}s^{p}\cdot \frac{1}{x^{p}}\;,$$

where ≈ denotes asymptotic equality in the limit x→∞, and where $\tau_{p}$ is a tail coefficient (namely, $\tau_{p}$ is a positive number that depends on the Levy exponent *p* alone).

Now, as in Section 4.1 and Section 4.2, consider the increment $X\left(t+\Delta \right)-X\left(t\right)$. When the Levy exponent is in the range 0<p<2, the increment’s tail-to-tail ratio is that of the following quantities: the numerator is the right-hand side of Equation (16)—with regard to the increment’s conditional distribution; and the denominator is the right-hand side of Equation (16)—with regard to the increment’s unconditional distribution. In turn, the tail-to-tail ratio is the *p*th power of the scale-to-scale ratio—that of the scale of the increment’s conditional distribution to the scale of the increment’s unconditional distribution.

So, the tail-to-tail ratio is the *p*th power of the noise-to-noise ratio of Equation (15): $1/[1+\phi_{p}\left(\alpha \Delta \right)]$. Similarly to the noise-to-noise ratio of Equation (15), the tail-to-tail ratio also takes values in the unit interval: it is bounded from below by the value zero, and it is bounded from above by the value one.

**Figure 3:**
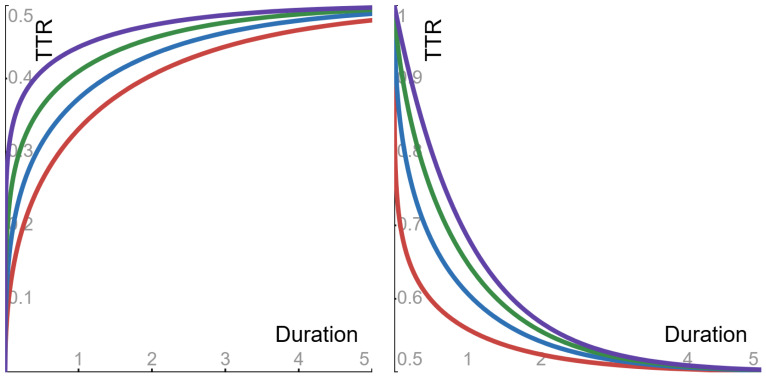
The tail-to-tail ratio (TTR), as a function of the duration Δ (with parameter α = 1). Sub-Cauchy examples are depicted in the **left panel**, with the following Levy exponents: red 0.5; blue 0.6; green 0.7; purple 0.8. Super-Cauchy examples are depicted in the **right panel**, with the following Levy exponents: red 1.25; blue 1.5; green 1.75; purple 1.99.

The properties of the function $\phi_{p}\left(v\right)$ were described in Section 4.1. Due to these properties, the asymptotic value—as Δ→∞—of the tail-to-tail ratio is 1/2. Also—as a function of the duration Δ—the tail-to-tail ratio displays the following behaviors (see Figure 3).

▶ **Sub-Cauchy** (0<p<1) **case**: the ratio is *monotone increasing* from 0 to its asymptotic value.
▶ **Cauchy** (p=1) **case**: the ratio is *flat*, and its constant value is its asymptotic value.
▶ **Super-Cauchy** (1<p<2) **case**: the ratio is *monotone decreasing* from 1 to its asymptotic value.

As noted in Section 2.4, the parameter α is the damping coefficient of the Langevin Equation (6), and it is positive. It follows straightforwardly that the monotonicity behavior of the tail-to-tail ratio $1/[1+\phi_{p}\left(\alpha \Delta \right)]$—with respect to the duration Δ—holds identically also with respect to the parameter α.

## 5. Gauss Approach

As described in the introduction, the OUP is a ‘regular’ stochastic model, and there are two main approaches that turn it to an ‘anomalous’ stochastic model: Levy and Gauss. The Levy approach was addressed in the sections above. This section addresses the Gauss approach.

Being a Gaussian stationary process (with zero means), the OUP is characterized by its auto-correlation function—which is exponential. Specifically, the correlation of the OUP positions $X\left(t\right)$ and $X\left(t+\Delta \right)$ is $\rho_{OUP}\left(\Delta \right)=\exp\left(-\alpha \Delta \right)$, where the exponent α is the positive damping coefficient of the Langevin Equation (6). Evidently, the OUP auto-correlation function $\rho_{OUP}\left(\Delta \right)$ (Δ≥0) is monotone decreasing from $\rho_{OUP}\left(0\right)=1$ to $\lim_{\Delta \to \infty }\rho_{OUP}\left(\Delta \right)=0$.

In the Gauss approach, the OUP is replaced by a Gaussian stationary process (with zero means) that is characterized by an auto-correlation function $\rho \left(\Delta \right)$ (Δ≥0). Akin to the exponential auto-correlation function of the OUP, the auto-correlation function $\rho \left(\Delta \right)$ is considered to be monotone decreasing from $\rho \left(0\right)=1$ to $\lim_{\Delta \to \infty }\rho \left(\Delta \right)=0$. The monotonicity of the auto-correlation function—which holds for the OUP, and which is set in the Gauss approach—is a natural assumption. Indeed, as $\rho \left(\Delta \right)$ is the correlation of the positions at the time points *t* and t+Δ, it is natural to assume the following: (**i**) the greater the temporal gap between the two time points, the lesser the correlation of the corresponding positions; and (**ii**) when the temporal gap grows infinitely large, the correlation decays to zero.

With regard to a process that is specified by the Gauss approach, the following results are proved in Appendix A. As with the OUP, the positions of the process are real-valued, and the position at the time point *t* is denoted $X\left(t\right)$.

**Signal-to-noise ratio**. The counterpart of the modified signal-to-noise ratio of Equation (14) is

(17) $$\frac{\left|M\left[X\left(t+\Delta \right)-X\left(t\right)|X\left(t\right)\right]\right|}{\sigma \left[X\left(t+\Delta \right)-X\left(t\right)|X\left(t\right)\right]}=\underset{\omega }{\underset{︸}{\left[\frac{\left|X\left(t\right)\right|}{\sigma_{2}\sqrt{V}}\right]}}\cdot \sqrt{\frac{2}{1+\rho \left(\Delta \right)}-1}\;,$$

where: the coefficient $\sigma_{2}$ is that of Equation (4); and *V* is the positions’ variance. In turn, the monotonicity of the auto-correlation function $\rho \left(\Delta \right)$ implies that (when the given information is not zero, $X\left(t\right)\neq 0$): as a function of the duration Δ, the signal-to-noise ratio of Equation (17) is *monotone increasing* from 0 to ω.

**Noise-to-noise ratio**. The counterpart of the noise-to-noise ratio of Equation (15) is

(18) $$\frac{\sigma \left[X\left(t+\Delta \right)-X\left(t\right)|X\left(t\right)\right]}{\sigma \left[X\left(t+\Delta \right)-X\left(t\right)\right]}=\sqrt{\frac{1+\rho \left(\Delta \right)}{2}}\;.$$

In turn, the monotonicity of the auto-correlation function $\rho \left(\Delta \right)$ implies that: as a function of the duration Δ, the noise-to-noise ratio of Equation (18) is *monotone decreasing* from 1 to $1/\sqrt{2}$.

**Tail-to-tail ratio**. For any positive duration Δ, the tail-to-tail ratio is *zero*.

The shapes noted above—increasing signal-to-noise ratio, decreasing noise-to-noise ratio, and zero tail-to-tail ratio—are invariant with respect to the auto-correlation function $\rho \left(\Delta \right)$. Thus, in particular, these shapes hold for the OUP.

## 6. Discussion

The three ratios that were devised in Section 4 quantify—each from its own perspective—the extent to which the information $X\left(t\right)$ affects the statistics of the increment $X\left(t+\Delta \right)-X\left(t\right)$. In other words, these ratios quantify the **memory** of the process under consideration: the Levy-driven OUP in Section 4; and the Gaussian stationary process (as specified by the Gauss approach) in Section 5.

This section concludes with a discussion of the results established in Section 4 and Section 5, and of the results’ counter-intuitive nature. The discussion addresses the following issues: Levy vs. Gauss comparison (Section 6.1); the Cauchy threshold (Section 6.2); Noah vs. Joseph comparison (Section 6.3); and Levy fluctuations (Section 6.4).

### 6.1. Levy vs. Gauss

The main results of Section 4 and Section 5—regarding the monotonicity of the three ratios with respect to the positive duration Δ—are summarized in Table 1. As evident from this table, the Levy approach yields **memory effects**, whereas the Gauss approach does *not*. Indeed, in the Gauss approach, each ratio displays a *single* type of behavior—which is identical for the OUP on the one hand, and for the Gaussian stationary process on the other hand. In sharp contrast, in the Levy approach each ratio displays *three different* types of behavior.

In the space of real-valued stationary processes, the OUP can be pictured as a ‘junction’ from which the two approaches fork out. The Levy approach is parameterized by the Levy exponent, 0<p≤2, and hence it has a single degree of freedom. The Gauss approach is parameterized by the monotone decreasing auto-correlation function, $\rho \left(\Delta \right)$ (Δ≥0), and hence it has infinitely many degrees of freedom.

Reasonably, one would expect that the Gauss approach (with its infinitely many degrees of freedom) generates richer statistical behaviors than the Levy approach (with its single degree of freedom). Yet, from the perspectives of the three ratios, the very converse holds. Indeed, the following counter-intuitive Levy vs. Gauss conclusion is attained: **the Levy approach generates richer statistical behaviors than the Gauss approach**.

### 6.2. Cauchy Threshold

The Levy exponent p=1, which characterizes the special case of the Cauchy distribution and the Cauchy noise, was termed ‘Cauchy threshold’ in Section 2. Indeed, as explained in that section, the mean behavior of the Levy distribution changes dramatically as the Levy exponent crosses the Cauchy threshold, and an intrinsic convexity of the Levy noise emerges/vanishes as the Levy exponent crosses the Cauchy threshold.

As evident from Table 1, the Levy exponent p=1 assumes a ‘threshold role’ also with regard to the three ratios: signal-to-noise, noise-to-noise, and tail-to-tail. Indeed, for each of these ratios, the monotonicity changes dramatically as the Levy exponent crosses the Cauchy threshold. For the signal-to-noise ratio and the noise-to-noise ratio, the following holds: above the Cauchy threshold, the monotonicity is identical to that of the OUP; and below the Cauchy threshold, the monotonicity flips, and it is antithetical to that of the OUP.

### 6.3. Noah vs. Joseph

The Levy approach and the Gauss approach ‘upgrade’ the OUP in two ‘orthogonal directions’: the *Noah effect* in the former, and the *Joseph effect* in the latter. The orthogonality of these directions shall now be explained and discussed.

In the Levy approach, the driving noise is changed from GWN to Levy noise. In turn, the statistics of the positions change from Gauss to Levy, i.e., from finite variances and light tails to infinite variances and heavy tails [46,47]. Mandelbrot and Wallis termed this change “Noah effect” [48]. Note that this change affects amplitudinal fluctuations in the noise, and it does not affect temporal dependencies of the noise. Indeed, both GWN and Levy noise share the **L2** Levy property of Section 2.3: the integrals of the noise over disjoint time intervals are independent random variables.

In the Gauss approach, the auto-correlation function is changed from exponential (which characterizes the OUP) to general (which is monotone decreasing to zero). In turn, the stationary correlation structure may change from short-range to long-range [123,124,125]. Namely, for a correlation structure that is determined by the auto-correlation function $\rho \left(\Delta \right)$ (Δ≥0): ‘short-range’ is when the auto-correlation is integrable at infinity, $\int_{1}^{\infty }\left|\rho \left(\Delta \right)\right|d\Delta <\infty$; and ‘long-range’ is when the auto-correlation is not integrable at infinity, $\int_{1}^{\infty }\left|\rho \left(\Delta \right)\right|d\Delta =\infty$. Mandelbrot and Wallis termed this change “Joseph effect” [48]. Note that this change affects temporal dependencies of the process, and it does not affect amplitudinal fluctuations of the process. Indeed, the statistics of the positions remain Gauss, and hence their variances remain finite and their tails remain ‘light’.

Reasonably, one would expect that the Noah effect will not affect the process memory, whereas the Joseph effect will. Yet, from the perspectives of the three ratios, the very converse holds. Indeed, the Levy vs. Gauss conclusion (stated in Section 6.1) further yields the following counter-intuitive Noah vs. Joseph conclusion: **the Noah effect affects process memory, whereas the Joseph effect does not**.

### 6.4. Levy Fluctuations

As noted in the opening of Section 4, the parameter of the function $\phi_{p}\left(v\right)$ (of Equation (13)) is the Levy exponent 0<p≤2. In addition, as established in Section 4, the three ratios of that section involve the function $\phi_{p}\left(v\right)$. Now, keep the variable *v* fixed, and vary the Levy exponent *p*.

**Figure 4:**
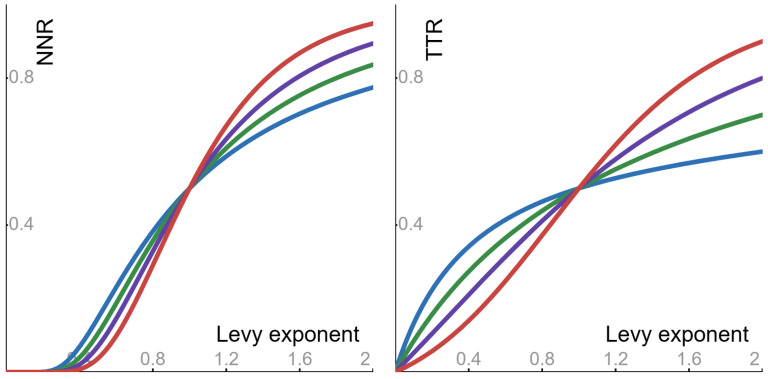
The noise-to-noise ratio (NNR) of Equation (15) and the tail-to-tail ratio (TTR), as functions of the Levy exponent *p* (with parameter α = 1). The examples are depicted with the following values of the duration Δ: blue −ln(0.2); green −ln(0.4); purple −ln(0.6); red −ln(0.8).

It is shown in Appendix A that—with respect to the Levy exponent *p*—the shape of $\phi_{p}\left(v\right)$ is as follows: it is monotone decreasing from the limit value $\lim_{p\to 0}\phi_{p}\left(v\right)=\infty$ to the value $\phi_{2}\left(v\right)=\tanh\left(\frac{1}{2}v\right)$. In turn, the noise-to-noise ratio of Equation (15) and the tail-to-tail ratio of Section 4.3 display the behaviors stated below (see Figure 4). The explanation why the shape of $\phi_{p}\left(v\right)$ implies the behavior of the noise-to-noise ratio is detailed in Appendix A.

▶ As a function of the Levy exponent *p*, the noise-to-noise ratio is *monotone increasing* from 0 to $\sqrt{\frac{1}{2}\left[1+\exp\left(-\alpha \Delta \right)\right]}$.
▶ As a function of the Levy exponent *p*, the tail-to-tail ratio is *monotone increasing* from 0 to $\frac{1}{2}\left[1+\exp\left(-\alpha \Delta \right)\right]$.

The probability tails of the Levy distribution were described in Equation (16). The following fact is evident from Equation (16): the smaller the Levy exponent *p*, the ‘heavier’ the probability tails—and hence the ‘wilder’ the fluctuations in the Levy noise.

As noted in Section 4.2, the noise-to-noise ratio of Equation (15) quantifies the extent to which the given information $X\left(t\right)$ reduces the measured randomness of the increment $X\left(t+\Delta \right)-X\left(t\right)$. Reasonably, one would expect that the ‘wilder’ the fluctuations in the Levy noise, the lesser the reduction in randomness. However, the behavior of the noise-to-noise ratio asserts the very converse: the smaller the Levy exponent *p*, the smaller the noise-to-noise ratio. So, the following counter-intuitive fluctuations conclusion is attained: **the wilder the fluctuations in the Levy noise, the greater the reduction in randomness**.

## Figures and Tables

**Table 1:** Levy approach vs. Gauss approach. The table’s rows correspond to the three ratios: signal-to-noise, noise-to-noise, and tail-to-tail. The table’s columns correspond, respectively, to the two approaches: the Levy-driven OUP, where the Levy exponent is in the ‘pure Levy’ range 0<p<2; and a Gaussian stationary process (with zero means), whose auto-correlation function is monotone decreasing to zero. The table’s cells describe the monotonicity of the ratios with respect to the positive duration Δ.

Ratio	Levy Approach	Gauss Approach
**Signal-to-noise**	$\begin{array}{c} \mathrm{decreasing}\;(p<1)\\ \mathrm{flat}\;(p=1)\\ \mathrm{increasing}\;(p>1) \end{array}$	increasing
**Noise-to-noise**	$\begin{array}{c} \mathrm{increasing}\;(p<1)\\ \mathrm{flat}\;(p=1)\\ \mathrm{decreasing}\;(p>1) \end{array}$	decreasing
**Tail-to-tail**	$\begin{array}{c} \mathrm{increasing}\;(p<1)\\ \mathrm{flat}\;(p=1)\\ \mathrm{decreasing}\;(p>1) \end{array}$	zero

## Data Availability

No new data were created or analyzed in this study.

## Appendix A

### Appendix A.1. The Function of Equation (13)

As noted in the opening of Section 4: the function of Equation (13) is $\phi_{p}\left(v\right)={\left[1-\exp\left(-v\right)\right]}^{p}/\left[1-\exp\left(-pv\right)\right]$; its variable is positive, 0<v<∞; and its parameter is the Levy exponent, 0<p≤2.

#### Appendix A.1.1. The Function of Equation (13) with Respect to Its Variable

It follows from Equation (13) that

(A1) $$\phi_{p}\left(v\right)=\frac{{\left[1-\exp\left(-v\right)\right]}^{p}}{1-\exp\left(-pv\right)}=\frac{{\left[\frac{1-\exp\left(-v\right)}{v}\right]}^{p}}{\frac{1-\exp\left(-pv\right)}{pv}}\cdot \frac{1}{p}v^{p-1}\;.$$

In turn, as $\lim_{x\to 0}\frac{1-\exp\left(-x\right)}{x}=1$, Equation (A1) implies that

(A2) $$\lim_{v\to 0}\phi_{p}\left(v\right)=\left\{\begin{array}{ccc} \infty & \; & \mathrm{when}\;0<p<1\;,\\ \; & \; & \\ 0 & \; & \mathrm{when}\;1<p\leq 2\;. \end{array}\right.$$

Setting $u=\exp\left(-v\right)$ implies that

(A3) $$\phi_{p}\left(v\right)=\frac{{\left(1-u\right)}^{p}}{1-u^{p}}\;.$$

In turn, Equation (A3) implies that

(A4) $$\left.\begin{array}{c} \frac{d\phi_{p}\left(v\right)}{dv}=\frac{-p{\left(1-u\right)}^{p-1}\left(1-u^{p}\right)+{\left(1-u\right)}^{p}pu^{p-1}}{{\left(1-u^{p}\right)}^{2}}\cdot \frac{du}{dv}\\ \;\\ =\frac{p{\left(1-u\right)}^{p-1}}{{\left(1-u^{p}\right)}^{2}}\left[u^{p}-1+\left(1-u\right)u^{p-1}\right]\cdot \left(-u\right)\\ \;\\ =\frac{p{\left(1-u\right)}^{p-1}u}{{\left(1-u^{p}\right)}^{2}}\left(1-u^{p-1}\right)\;. \end{array}\right.$$

As the variable *v* is positive, the variable *u* takes values in the unit interval: 0<u<1. Consequently, Equation (A4) implies that:

(A5) $$\left.\begin{array}{c} \frac{d\phi_{p}\left(v\right)}{dv}<0\;\;\;\mathrm{when}\;\;\;0<p<1\;,\\ \;\\ \frac{d\phi_{p}\left(v\right)}{dv}>0\;\;\;\mathrm{when}\;\;\;1<p\leq 2\;. \end{array}\right.$$

#### Appendix A.1.2. The Function of Equation (13) with Respect to Its Parameter

It follows from Equation (13) that

(A6) $$\phi_{p}\left(v\right)=\frac{{\left[1-\exp\left(-v\right)\right]}^{p}}{\frac{1-\exp\left(-pv\right)}{pv}}\cdot \frac{1}{pv}$$

In turn, as $\lim_{p\to 0}{\left[1-\exp\left(-v\right)\right]}^{p}=1$ and as $\lim_{x\to 0}\frac{1-\exp\left(-x\right)}{x}=1$, Equation (A6) implies that

(A7) $$\lim_{p\to 0}\phi_{p}\left(v\right)=\infty$$

Equation (A3) implies that

(A8) $$\left.\begin{array}{c} \frac{\partial \phi_{p}\left(v\right)}{\partial p}=\frac{{\left(1-u\right)}^{p}\ln\left(1-u\right)\left(1-u^{p}\right)+{\left(1-u\right)}^{p}u^{p}\ln\left(u\right)}{{\left(1-u^{p}\right)}^{2}}\\ \;\\ =\frac{{\left(1-u\right)}^{p}}{{\left(1-u^{p}\right)}^{2}}\left[\ln\left(1-u\right)\left(1-u^{p}\right)+\ln\left(u\right)u^{p}\right]\;. \end{array}\right.$$

As noted above, the variable *u* takes values in the unit interval: 0<u<1. Consequently, $\ln\left(1-u\right)<0$ and $\ln\left(u\right)<0$, and hence

(A9) $$\frac{\partial \phi_{p}\left(v\right)}{\partial p}<0\;.$$

#### Appendix A.1.3. A Transformation of the Function of Equation (13)

Consider the function

(A10) $$\psi_{p}\left(v\right)=\frac{1}{{\left[1+\phi_{p}\left(v\right)\right]}^{1/p}}\;,$$

where $\phi_{p}\left(v\right)$ is the function of Equation (13), and (as above): the variable is positive, 0<v<∞; and the parameter is the Levy exponent, 0<p≤2. Equation (A10) implies that

(A11) $$\ln\left[\psi_{p}\left(v\right)\right]=-\frac{1}{p}\ln\left[1+\phi_{p}\left(v\right)\right]\;.$$

In turn, Equation (A11) implies that

(A12) $$\left.\begin{array}{c} \frac{\frac{\partial \psi_{p}\left(v\right)}{\partial p}}{\psi_{p}\left(v\right)}=\frac{\partial \ln\left[\psi_{p}\left(v\right)\right]}{\partial p}\\ \;\\ =\frac{1}{p^{2}}\ln\left[1+\phi_{p}\left(v\right)\right]-\frac{1}{p}\frac{\frac{\partial \phi_{p}\left(v\right)}{\partial p}}{1+\phi_{p}\left(v\right)}\;. \end{array}\right.$$

As $\phi_{p}\left(v\right)$ is positive, $\ln\left[1+\phi_{p}\left(v\right)\right]>0$. Consequently, Equations (A9) and (A12) imply that

(A13) $$\frac{\partial \psi_{p}\left(v\right)}{\partial p}>0\;.$$

### Appendix A.2. Bivariate Normal Calculations

Consider a random vector $\left(Z_{1},Z_{2}\right)$ whose statistical distribution is bivariate normal [6,7,8] with the following characteristics. (**I**) Zero means: $E\left[Z_{1}\right]=0$ and $E\left[Z_{2}\right]=0$. (**II**) Identical variances: $\mathrm{Var}\left[Z_{1}\right]=v$ and $\mathrm{Var}\left[Z_{2}\right]=v$, where *v* is a positive number. (**III**) Covariance $\mathrm{Cov}\left[Z_{1},Z_{2}\right]=rv$, where *r* is a correlation that is smaller than one: r<1 (this condition means that the components of the random vector $\left(Z_{1},Z_{2}\right)$ are not perfectly correlated). In addition, consider a general measure of randomness σ[·] (as described in Section 2.1).

#### Appendix A.2.1. Signal-to-Noise Ratio

The conditional statistical distribution of $Z_{2}$—given the information $Z_{1}$—is normal with the following characteristics [6,7,8]: conditional mean

(A14) $$E\left[Z_{2}|Z_{1}\right]=rZ_{1};$$

and conditional variance

(A15) $$\mathrm{Var}\left[Z_{2}|Z_{1}\right]=\left(1-r^{2}\right)v\;.$$

In turn, the conditional statistical distribution of the difference $\left(Z_{2}-Z_{1}\right)$—given the information $Z_{1}$—is normal with the following characteristics: conditional mean

(A16) $$E\left[Z_{2}-Z_{1}|Z_{1}\right]=\left(r-1\right)Z_{1};$$

and conditional variance

(A17) $$\mathrm{Var}\left[Z_{2}-Z_{1}|Z_{1}\right]=\left(1-r^{2}\right)v\;.$$

As the mean and the median of the normal statistical distribution coincide, Equation (A16) implies that

(A18) $$M\left[Z_{2}-Z_{1}|Z_{1}\right]=\left(r-1\right)Z_{1}.$$

Equations (4) and (A17) (together with the fact that the variance is the square of the standard deviation) imply that

(A19) $$\sigma \left[Z_{2}-Z_{1}|Z_{1}\right]=\sigma_{2}\sqrt{\left(1-r^{2}\right)v}\;,$$

where the coefficient $\sigma_{2}$ is that of Equation (4). In turn, Equations (A18) and (A19) yield the following signal-to-noise ratio:

(A20) $$\frac{\left|M\left[Z_{2}-Z_{1}|Z_{1}\right]\right|}{\sigma \left[Z_{2}-Z_{1}|Z_{1}\right]}=\frac{\left|Z_{1}\right|}{\sigma_{2}\sqrt{v}}\frac{1-r}{\sqrt{1-r^{2}}}=\frac{\left|Z_{1}\right|}{\sigma_{2}\sqrt{v}}\sqrt{\frac{2}{1+r}-1}\;.$$

#### Appendix A.2.2. Noise-to-Noise Ratio

The statistical distribution of the difference $\left(Z_{2}-Z_{1}\right)$ is normal with the following characteristics: mean

(A21) $$E\left[Z_{2}-Z_{1}\right]=E\left[Z_{2}\right]-E\left[Z_{1}\right]=0;$$

and variance

(A22) $$\left.\begin{array}{c} \mathrm{Var}\left[Z_{2}-Z_{1}\right]\\ \;\\ =\mathrm{Var}\left[Z_{1}\right]+\mathrm{Var}\left[Z_{2}\right]-2\mathrm{Cov}\left[Z_{1},Z_{2}\right]\\ \;\\ =2v-2rv=(1-r)2v\;. \end{array}\right.$$

Consequently, Equations (A17) and (A22) yield the following variance-to-variance ratio:

(A23) $$\frac{\mathrm{Var}\left[Z_{2}-Z_{1}|Z_{1}\right]}{\mathrm{Var}\left[Z_{2}-Z_{1}\right]}=\frac{\left(1-r^{2}\right)v}{(1-r)2v}=\frac{1+r}{2}\;.$$

In turn, Equations (4) and (A23) (together with the fact that the variance is the square of the standard deviation) yield the following noise-to-noise ratio

(A24) $$\frac{\sigma \left[Z_{2}-Z_{1}|Z_{1}\right]}{\sigma \left[Z_{2}-Z_{1}\right]}=\sqrt{\frac{1+r}{2}}\;.$$

#### Appendix A.2.3. Tail-to-Tail Ratio

With regard to the difference $\left(Z_{2}-Z_{1}\right)$, use the following shorthand notation: $a^{2}$ is the conditional variance of Equation (A17); and $b^{2}$ is the variance of Equation (A22). Note that

(A25) $$b^{2}-a^{2}=\left(1-r\right)2v-\left(1-r^{2}\right)v={\left(1-r\right)}^{2}v\;.$$

As the correlation is smaller than one, r<1, the difference of Equation (A25) is positive: $b^{2}-a^{2}>0$.

The probability that the absolute value of a ‘standard’ normal random variable (i.e., with mean zero and with variance one) is greater than the positive level *l* is

(A26) $$\Phi \left(l\right):=\int_{l}^{\infty }\sqrt{\frac{2}{\pi }}\exp\left(-\frac{1}{2}u^{2}\right)du\;.$$

In turn, the probability that the deviation of a general normal random variable from its mean is greater than the positive number *x* is $\Phi \left(x/s\right)$, where *s* is the standard deviation of the general normal random variable.

So, with regard to the conditional statistical distribution of the difference $\left(Z_{2}-Z_{1}\right)$, given the information $Z_{1}$, Equations (A16) and (A17) imply that

(A27) $$\mathrm{Pr}\left[\left|\left(Z_{2}-Z_{1}\right)-\left(r-1\right)Z_{1}\right|>x|Z_{1}\right]=\Phi \left(\frac{x}{a}\right)\;.$$

With regard to the (unconditional) statistical distribution of the difference $\left(Z_{2}-Z_{1}\right)$, Equations (A21) and (A22) imply that

(A28) $$\mathrm{Pr}\left[\left|\left(Z_{2}-Z_{1}\right)-0\right|>x\right]=\Phi \left(\frac{x}{b}\right)\;.$$

L’Hopital’s rule and Equation (A26) imply that

(A29) $$\left.\begin{array}{c} \lim_{x\to \infty }\frac{\Phi \left(\frac{x}{a}\right)}{\Phi \left(\frac{x}{b}\right)}=\lim_{x\to \infty }\frac{\Phi^{\prime }\left(\frac{x}{a}\right)\frac{1}{a}}{\Phi^{\prime }\left(\frac{x}{b}\right)\frac{1}{b}}\\ \;\\ =\frac{b}{a}\lim_{x\to \infty }\frac{\exp\left[-\frac{1}{2}{\left(\frac{x}{a}\right)}^{2}\right]}{\exp\left[-\frac{1}{2}{\left(\frac{x}{b}\right)}^{2}\right]}\\ \;\\ =\frac{b}{a}\lim_{x\to \infty }\exp\left\{-\frac{1}{2}\frac{b^{2}-a^{2}}{a^{2}b^{2}}x^{2}\right\}\;. \end{array}\right.$$

In turn, as $b^{2}-a^{2}>0$, Equation (A29) yields the following tail-to-tail ratio:

(A30) $$\lim_{x\to \infty }\frac{\Phi \left(\frac{x}{a}\right)}{\Phi \left(\frac{x}{b}\right)}=0\;.\;$$

#### Appendix A.2.4. Gaussian Stationary Processes

Consider a Gaussian stationary process—as specified in the ‘Gauss direction’ of Section 5—with a monotone-decreasing auto-correlation function $\rho \left(\Delta \right)$ (Δ≥0). Further consider the increment $X\left(t+\Delta \right)-X\left(t\right)$ of the Gaussian stationary process. Using the above notation, set the components of the bivariate mormal random vector $\left(Z_{1},Z_{2}\right)$ to be the following: $Z_{1}=X\left(t\right)$ and $Z_{2}=X\left(t+\Delta \right)$. In turn, the corresponding correlation is $r=\rho \left(\Delta \right)$, and hence: Equation (A20) yields the signal-to-noise ratio of Equation (17), and Equation (A24) yields the noise-to-noise ratio of Equation (18). Also, Equation (A30) implies that the tail-to-tail ratio is zero.
